# Monoblock or modular? Impact of stem design and conicity angle on long-term implant survival in revision total hip arthroplasty: a 20-year follow-up registry study on 3647 implants

**DOI:** 10.1007/s00590-025-04616-7

**Published:** 2025-12-08

**Authors:** Matteo Brunello, Alberto Di Martino, Manuele Morandi, Claudio D’Agostino, Chiara Di Censo, Barbara Bordini, Cesare Faldini

**Affiliations:** 1 Department of Biomedical and Neurimotor Sciences - DIBINEM - University of Bologna, Bologna, Italy; 2 1st Orthopedic Department, IRCCS - Istituto Ortopedico Rizzoli, Bologna, Italy; 3 Medical Technology Laboratory, IRCCS - Istituto Ortopedico Rizzoli, Bologna, Italy

**Keywords:** Total hip arthroplasty, Total hip revision, Stem, Monoblock, Monolithic, Modular, Registry study

## Abstract

**Introduction:**

Revision total hip arthroplasty (rTHA) often requires femoral stem revision due to aseptic loosening, instability, or fractures. Long, tapered conical stems are preferred for stability and bone loss management. Recent design changes, like increased conicity angles, aim to enhance fixation and reduce subsidence. Monoblock and modular stems offer distinct pros and cons, but their long-term outcomes remain debated. This study evaluates the long-term survival of monoblock versus modular conical stems, with a focus on conicity angles (2° vs. 3°), using 20 years of registry data.

**Methods:**

A retrospective review was conducted using the Emilia Romagna Registry of Orthopedic Prosthetic Implants (RIPO) from 2000 to 2021. A total of 3,647 non-cemented conical stems used in rTHA were analyzed: 32.4% monoblock and 67.6% modular. Kaplan–Meier survival analysis assessed implant longevity, stratified by stem design and conicity angle.

**Results:**

Use of modular stems increased from 24% to over 83% during the study period. Overall failure rate was 6.3–5.1% for monoblock stems (mainly due to aseptic loosening) and 6.9% for modular stems (primarily due to instability). Monoblock stems had superior 20-year survival (93.7%) compared to modular (86.8%, *p* = 0.009). Among modular stems, those with 2° conicity had significantly better 15-year survival (91.9%) than 3° designs (88.0%, *p* = 0.001). No significant difference was observed between conicity angles in monoblock stems.

**Conclusion:**

Monoblock stems provide better long-term survival in rTHA. Modular designs, while offering intraoperative flexibility, carry higher revision risk, especially with greater conicity. Stem selection should balance design features with patient needs and surgical expertise.

## Introduction

 The rate of Revision Total Hip Arthroplasty (rTHA) is progressively rising alongside the global increase in primary total hip surgeries [1]. It is estimated that approximately 15% of THAs currently require revision surgery [2–5]. In nearly half of these cases, the femoral stem is revised, often due to aseptic loosening, periprosthetic fracture, infection, or implant instability [6]. The goal of revision surgery is to achieve primary stability despite the associated femoral bone loss, in many cases at the expenses of the use of a longer femoral implant [7]. Several femoral stem designs are available, with most revision implants featuring long, conical designs. Early revision implants were predominantly cylindrical, but this design was largely abandoned due to its association with higher rates of stress shielding and periprosthetic fractures [8, 9]. For these reasons tapered stems were developed, showing several advantages; at first, these conferred a uniform load transmission and an even stress distribution between the implant and the surrounding bone; secondly, due to the tapered conical design, surgeons could modify femoral anteversion reducing the risk of dislocation [10]. Primary stability in tapered conical stems is achieved through their shape, which confers longitudinal stability while longitudinal grooves enhance rotational fixation. Over time, the design evolved by increasing the conicity angle from 2° to 3° in an effort to improve axial stability [11]; however, this modification was associated with subsidence—a progressive sinking of the stem that alters hip biomechanics and that results in leg length discrepancy and instability, among the most common causes of re-revision [10, 12]. To address these issues, modular long tapered stems were developed, in which bone is prepared separately at the proximal and distal femoral interfaces; in this configuration, primary stability is attained through distal fixation, and the proximal body of the implant, available in various lengths and offsets, permits intraoperative optimisation of hip biomechanics, including offset restoration, leg length correction and adjustment of stem version [12]. Nonetheless, modular devices have several disadvantages, such as a higher risk of intraoperative fractures, fatigue fractures at the modular junction, corrosion and increased costs [13, 14]. Despite these advancements, there remains no clear consensus as to which stem design offers the best clinical outcomes and long-term survival, and so far no registry study ever compared the survival of revision stem implants comparing modularity and conicity angle.

Therefore, the aim of this study was to evaluate the long-term survival of monoblock and modular conical stems in revision THA, leveraging a large registry-based dataset. Specifically, the study sought to compare the demographic characteristics of patient cohorts, assessing which stem design provided superior long-term survival, identifying the primary causes of failure associated with each type of implant, and investigating the impact of conicity angle (2° vs. 3°) on clinical outcomes and implant stability.

## Materials and methods

A registry-based retrospective study was conducted reporting and analyzing data collected by the Emilia Romagna Registry of Orthopedic Prosthetic Implants (RIPO). Emilia Romagna (ER) is an Italian region with 4.5 million inhabitants, and RIPO reports data about hip, knee, and shoulder arthroplasty procedures performed in the Region. Founded in 1990, the registry has a capture rate of approximately 98% on the implants performed at all the local orthopedic departments, involving a total of 62 hospitals (both public and private). The specific design of the registry allows comparisons with other national registries worldwide.

The study focused on rTHA procedures performed between 2000 and 2021: all the patients treated by stem revision during rTHAs within this timeframe and officially registered in the RIPO registry were included in the study. There were no restrictions for patients’ inclusion based on age and gender. The study focused exclusively on patients residing within the ER Region to mitigate potential bias originated from loss at follow-up. As a result, any rTHA performed on patients residing outside ER were deliberately excluded from the analysis. Isolated cup revisions, hemiarthroplasties, resurfacing procedures, and the use of megaprostheses for neoplastic and non-neoplastic conditions were also excluded. Data extraction from the RIPO database was performed on January 9th 2025, and implant survival and failure were collected until December 31 st, 2021.

RIPO standard reporting includes stem manufacturer, implant model and fixation, but it does not specify the geometric shape categorization of stems. Therefore, two researchers (MB, MMG) independently categorized conical stem type for each implant model present in the RIPO database; therefore, a division into modular and monoblock types, and in terms of conicity angle (2° vs. 3° degree of conicity) was performed, extrapolating data from stem brochures. In case of disagreement, the senior author (ADM) determined the most appropriate stem attribution. The study considered several variables, including patients age at surgery, gender, BMI; the number of cementless femoral stems implanted in stem revision THAs during the study period was collected and categorized according to modular or not-modular components. Implant survival was analyzed for each stem type, with failure defined as any surgery requiring revision of at least the femoral stem.

The total number of stem failures requiring implant removal or revision during the study period was assessed for each conical type, along with the corresponding percentage relative to the total number of implanted stems for each group. The long-term survival rate of the implant was evaluated. All the complications leading to the failure of femoral stems were described: this comprehensive evaluation allowed to assess, for each conical type, the incidence and the magnitude of specific stem complications with respect to the total of causes of stem failure. Data related to implant failures due to isolated acetabular cup-related complications were not included in the current analysis to reduce potential bias in interpreting the results, while exclusively focusing on the failures of the femoral component.

Implants were divided into modular and monoblock. The stems used in the modular group were as follows: Revision Lima™ (LimaCorporate, Villanova, Italy) – *n* = 1120; Alata Aequa Revision™ (Adler-Ortho, Cormano, Italy) – *n* = 450; Restoration Stryker-Howmedica™ (Stryker, Kalamazoo, MI, USA) – *n* = 222; MP Reconstruction Link™ (Waldemar Link, Hamburg, Germany) – *n* = 181; Modulus Hip System Lima™ (LimaCorporate, Villanova, Italy) – *n* = 98; Alata Acuta S™ (Adler-Ortho, Italy) – *n* = 83; MGS Samo™ (Samobiomedica, Italy) – *n* = 71. For the monoblock group: SL Revision Sulzer Centerpulse Zimmer™ (Zimmer, Warsaw, IN, USA) – *n* = 957; ConeLock Revision™ (Biomet, Warsaw, IN, USA) – *n* = 127; Redapt™ (Smith & Nephew, London, UK) – *n* = 76.

Ethical approval was not required for this study, since data collection is a regional ER standard practice, and the identity of the patients is concealed. Furthermore, no adjunctive clinical procedures were performed besides the analysis of the registry data.

### Statistical analysis

Descriptive statistics, such as mean, median and range for continuous variables and frequency with percentage (%) for categorical variables were used for data report. The chi-square test was employed to assess statistical significance of qualitative data, while the T test was used for continuous data. A p-value < 0.05 was considered statistically significant. Kaplan–Meier survivorship analysis was performed using the revision of at least the femoral stem component as endpoint, with implant survival of non-revised THAs considered as the last date of observation (December 31 st, 2022, or the date of death available from the ER database). The log-rank test was used to compare survivorship between groups. Statistical analyses were conducted using SPSS 14.0, version 14.0.1 (SPSS Inc., Chicago, IL, USA), and JMP, version 12.0.1 (SAS Institute Inc., Cary, NC, USA, 1989–2007).

## Results

### What are the demographic characteristics of the patients included in the study, and how do they compare between monoblock and modular stem groups?

A total of 3647 stem revisions of THA by conical stems were performed in ER between 2000 and 2021 and registered in the RIPO registry. The demographic analysis revealed that the baseline characteristics of patients undergoing revision THA were well-balanced between the monoblock and modular stem groups. The median age in both cohorts was 75.0 years, with a range of 28.0–98.0 years in the monoblock group and 19.0–96.0 years in the modular group. The average ages were 73.4 ± 9.8 years for monoblock stems and 73.6 ± 10.4 years for modular stems, showing no significant difference between the two groups (*p* = 0.593). The distribution of age classes was also comparable, with 82.9% of patients aged 65 years or older in the monoblock group and 83.5% in the modular group (*p* = 0.673).

As regards sex distribution, a significantly higher proportion of females were present in the monoblock cohort (64.4%) compared to the modular cohort (60.2%; *p* = 0.017). BMI distribution was comparable across cohorts, showing no significant differences (*p* = 0.423) (Table 1).

**Table 1:** Demographic characteristics of study patients

Characteristic	Monoblock *N* = 1,182	Modular *N* = 2,465	*p*-value
Age			0.593
Median (Range)	75.0 (28.0, 98.0)	75.0 (19.0, 96.0)	
Mean (SD)	73.4 (9.8)	73.6 (10.4)	
Age class			0.673
< 65	202 (17.1)	406 (16.5)	
≥ 65	980 (82.9)	2,059 (83.5)	
Sex			**0.017**
F	761 (64.4)	1,484 (60.2)	
M	421 (35.6)	981 (39.8)	
BMI			0.423
Underweight	10 (1.0)	25 (1.3)	
Normal Weight	307 (31.1)	658 (33.5)	
Overweight	455 (46.1)	848 (43.2)	
Obese	216 (21.9)	433 (22.0)	
Unknown	194	501	

**Fig. 1:**
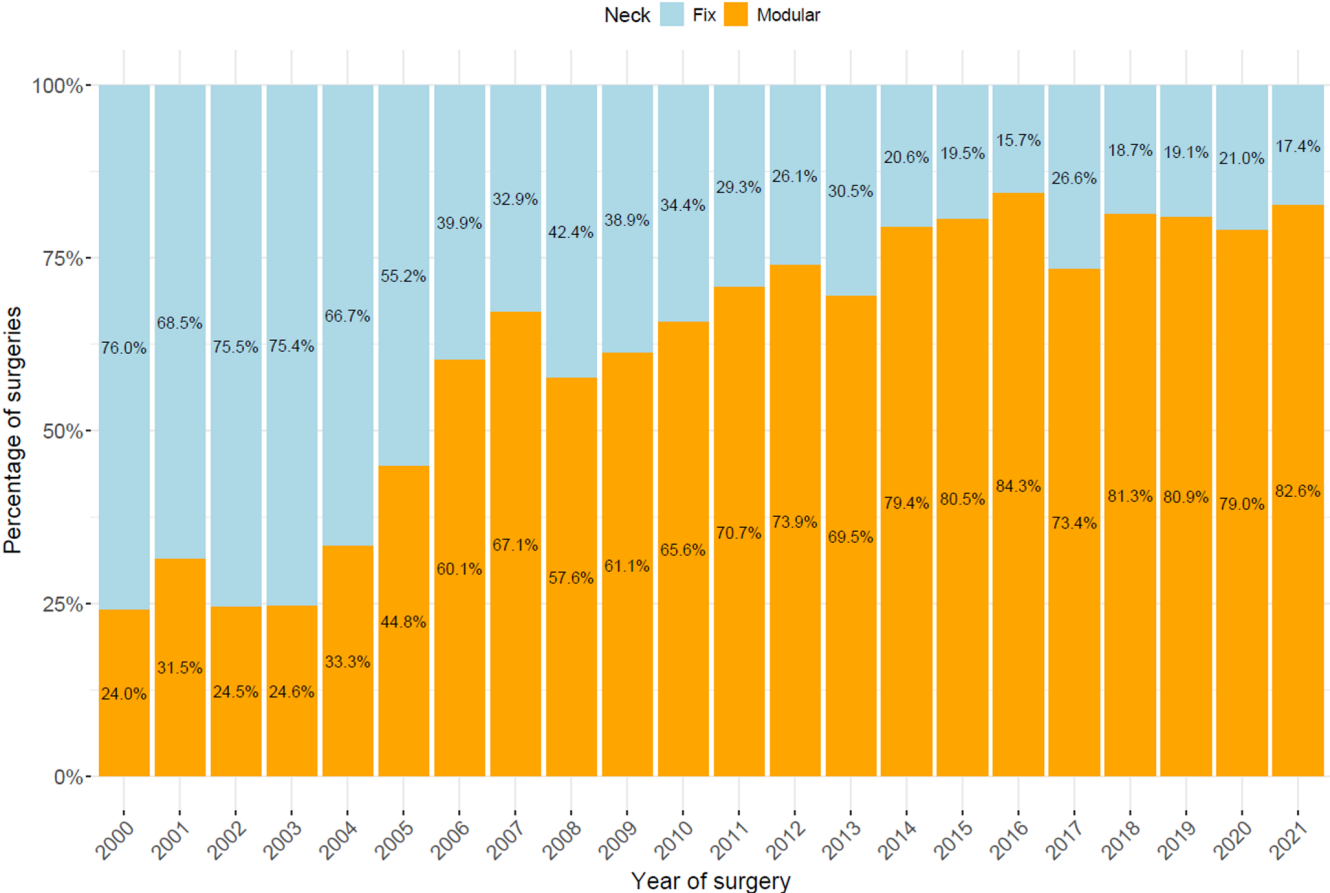
Evolution of distribution in the use of modular (orange) and monoblock (blue) stems during the study period

The yearly use of fixed and modular stem implants between 2000 and 2021 is available in Fig. 1; the trend in the use of tapered stems changed overtime in the 20 year timeframe, with the use of modular stems increasing from 24% in the early 2000 s to over 83% in more recent years.

### Which revision stem design (monoblock or modular) provides better long-term survival in rTHA?

**Fig. 2:**
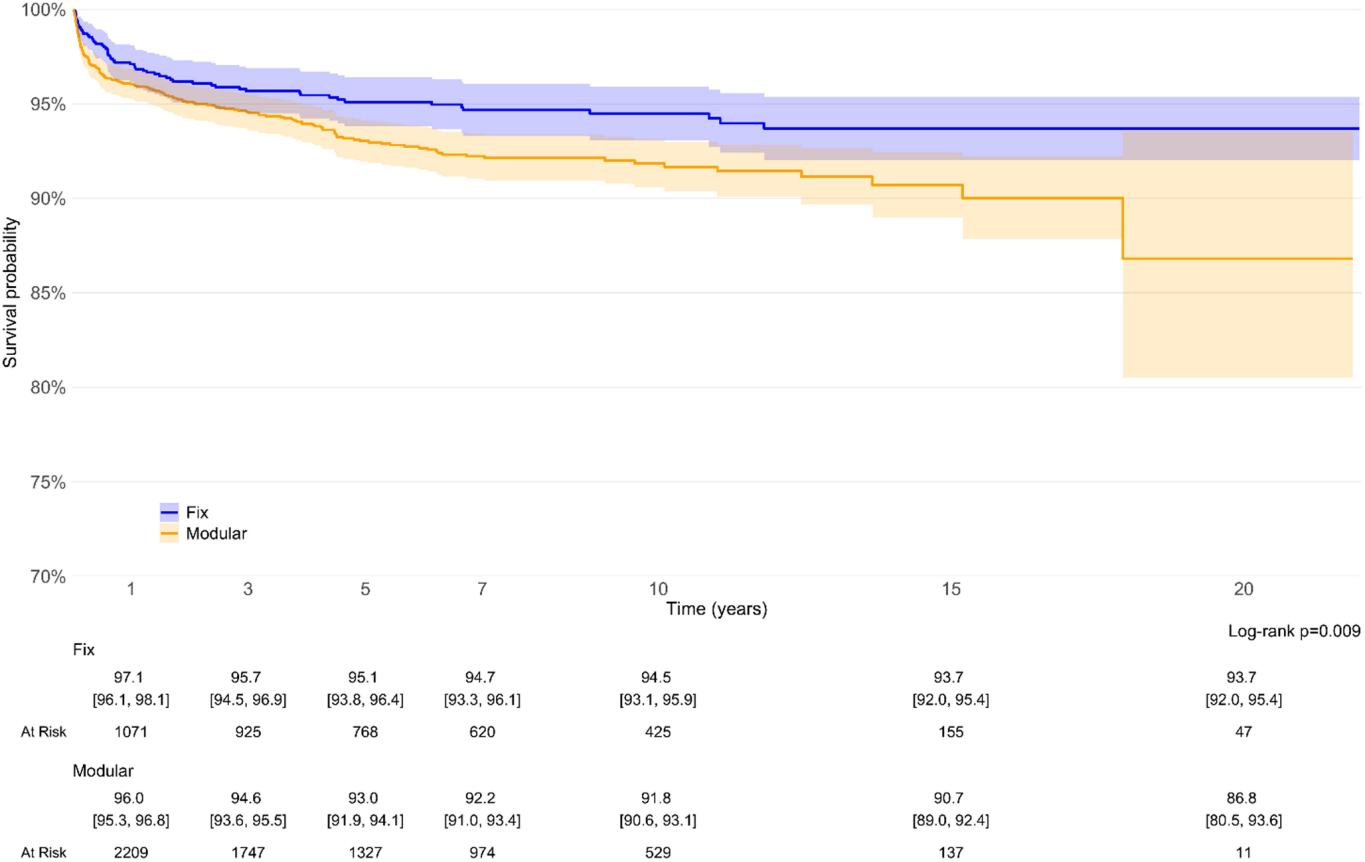
overall survival of modular (orange) vs. monoblock (purple) stems

Survival analysis demonstrated a superior long-term performance of monoblock conical stems compared to modular designs. Kaplan-Meier survivorship curves showed a 20-year survival probability of 93.7% (95% CI: 92.0–95.4%) for monoblock stems, significantly higher than the 86.8% (95% CI: 80.5–93.6%) observed for modular stems (*p* = 0.009). The survival superiority of monoblock stems was consistent at all follow-up intervals (Fig. 2).

### What are the main causes of failure associated with the two types of stems?

A total of 230 cases of failures were observed in the study population, with an overall failure rate of 6.3%; when data were divided by stem type, 5.1% of monoblock implants failed compared to 6.9% of modulars. The analysis highlighted distinct patterns of failure between monoblock and modular stem designs; in the monoblock group, stem aseptic loosening was the primary cause of revision, accounting for 31.7% of all failures (19/60 cases). Conversely, in the modular group, instability and dislocation represented the leading failure mechanism, responsible for 24.7% of failures (42/170 cases). Only one case of dislocation was reported among the 1,182 monoblock stems, compared to 42 cases in the modular group. Septic loosening was the second most common cause of failure in both groups, with a slightly higher rate encountered in monoblock stems (26.7%) compared to modular stems (20.6%). Periprosthetic fractures were rare in both cohorts, affecting only 0.2% of implants. Table 2 provides a breakdown of the number of failures during the 20-years study period according to stem types.

**Table 2:** Causes of failure associated with the two types of stems

	Monoblock, *N* = 1,182			Modular, *N* = 2,465		
Cause of failure	*N*	IR (%)	% cause fall.	*N*	IR (%)	% cause fall.
Septic loosening	16	1.4	26.7	35	1.4	20.6
Stem aseptic loosening	19	1.6	31.7	28	1.1	16.5
Dislocation/Instability	1	0.1	1.7	42	1.7	24.7
Unknown	16	1.4	26.7	19	0.8	11.2
Cup aseptic loosening	1	0.1	1.7	15	0.6	8.8
Implant rupture	1	0.1	1.7	8	0.3	4.7
*Neck breakage*				*1*		
*Neck and insert breakage*				*1*		
*Broken taproot cup*				*1*		
*Stem breakage*				*4*		
*Stem breakage + periprosthetic fracture*	*1*					
*Head breakage*				*1*		
Other	1	0.1	1.7	6	0.2	3.5
Early infection	1	0.1	1.7	6	0.2	3.5
Periprosthetic fracture	2	0.2	3.3	4	0.2	2.4
Global aseptic loosening		0.0	0.0	6	0.2	3.5
Dislocation + periprosthetic fracture	2	0.2	3.3	1	0.0	0.6
Total	60	5.1	100.0	170	6.9	100.0

### Does conicity angle (2° vs. 3°) influence long-term survival in revision THA?

**Fig. 3:**
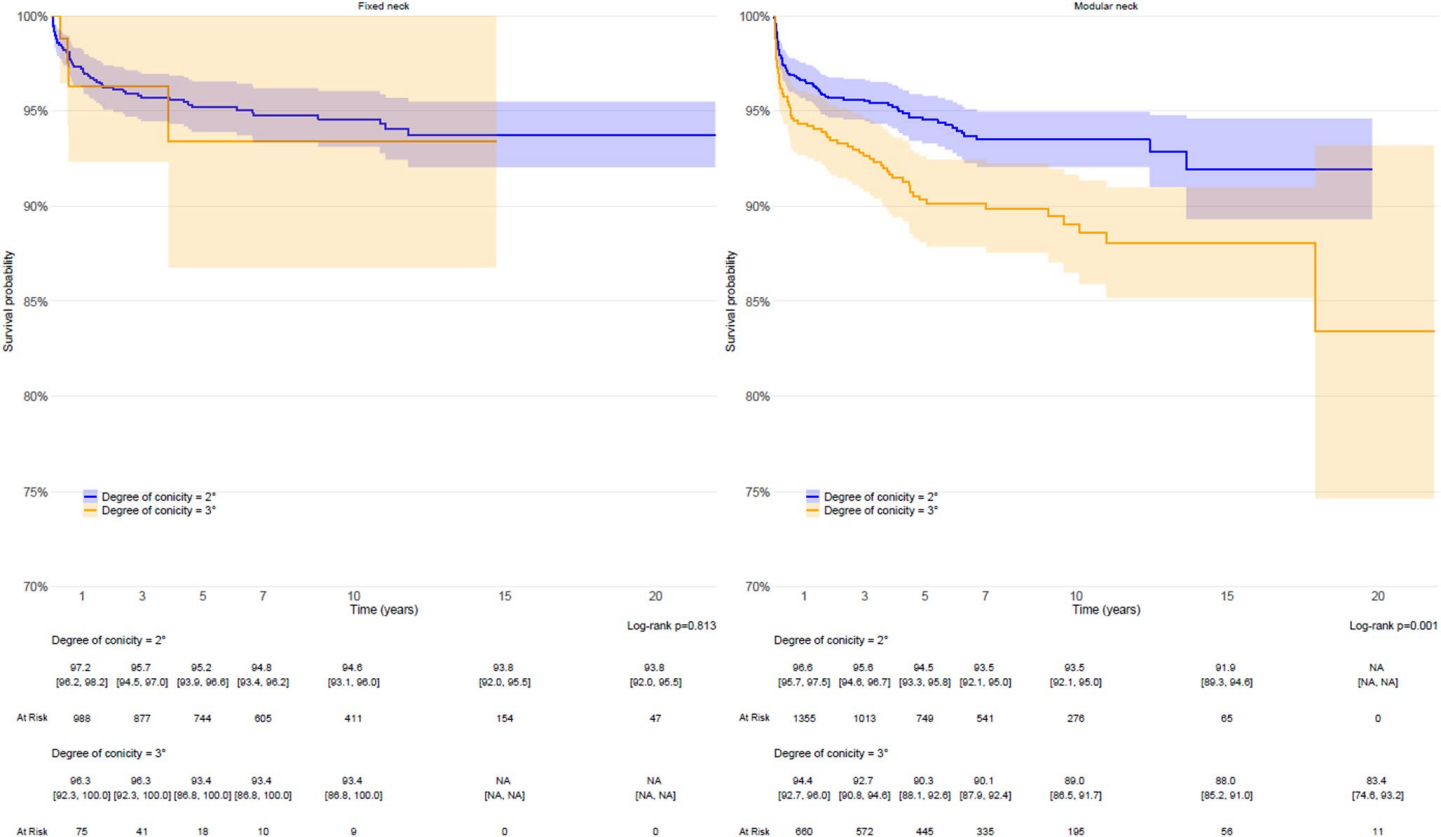
Kaplan–Meier survival analysis comparing femoral stem survival based on conicity angle (2° vs. 3°). Stems were analyzed separately for fixed neck and modular neck designs. In the fixed neck group, 1,084 stems (92.2%) had a 2° conicity and 89 stems (7.6%) had a 3° conicity. In the modular neck group, 1,523 stems (61.8%) had a 2° conicity and 740 stems (30.0%) had a 3° conicity. While no significant difference in long-term survival was observed for fixed neck stems (Log-rank *p* = 0.813), in the modular group a 3° conicity was associated with significantly lower survival (Log-rank *p* = 0.001).

 When analyzing implant survival within each group (monoblock and modular), the degree of conicity showed a different impact. In the monoblock group, no significant difference was found between stems with 2° and 3° conicity angles (*p* = 0.813), with both achieving high long-term survival rates. Monoblock stems with a 2° conicity angle showed a 20-year survival rate of 93.8% (95% CI: 92.0–95.5%), while those with a 3° angle presented slightly lower rates, especially after 10 years of follow-up. In contrast, modular stems with a 2° conicity angle had a 15-year survival rate of 91.9% (95% CI: 89.3–94.6%), significantly higher than the 88.0% (95% CI: 85.2–91.0%) observed in implants with 3° conicity angle (*p* = 0.001), indicating that higher conicity negatively affected implant longevity in modular designs (Fig. 3).

## Discussion

Findings of the current registry study show that long monoblock stems used in revision surgery had a significantly higher 20-year survival rate compared to modular stems (93.7% vs. 86.8%, *p* = 0.009). Conseguently, failure rate was lower for monoblock stems (5.1%) compared with modular stems (6.9%), suggesting that monoblock design offers superior long-term performance. An analysis of the causes of failure revealed distinct patterns comparing the two stem designs: in the monoblock group, aseptic loosening of the stem was the leading cause of failure (31.7% of failures), while in the modular group, instability and dislocation were predominant (24.7% of failures). Notably, only one case of dislocation/instability was recorded in the monoblock cohort compared with 42 cases in the modular group. The impact of conicity angle on implant survival varied depending on stem design: in monoblock stems, survival rates were high regardless of conicity, with no significant difference between 2° and 3° angles (*p* = 0.813). Specifically, stems with a 2° conicity angle reached a 20-year survival of 93.8% (95% CI: 92.0–95.5%), while those with 3° showed slightly reduced longevity, especially after the first decade. Conversely, in modular stems, conicity played a more critical role; a significant difference emerged between the two configurations (*p* = 0.001): stems with a 2° angle maintained better long-term performance, achieving a 15-year survival rate of 91.9% (95% CI: 89.3–94.6%), compared to 88.0% (95% CI: 85.2–91.0%) in those with a 3° angle.

The current study is subject to several limitations inherent to registry-based research, primarily due to its retrospective design and observational nature [15]. Consequently, our findings can only reveal associations between variables, which do not necessarily imply causality. Additionally, patients who underwent THA re-revision in regions outside Emilia-Romagna were lost to follow-up and therefore not captured, potentially underestimating true failure rates. Furthermore, certain critical data—such as radiographic images, quantitative measurements of subsidence, bone defect severity, and acetabular status—were not available from the registry. Likewise, clinical scores (HHS, HOOS) and functional outcomes were not recorded, restricting the analysis exclusively to revision-free survival. Another limitation lies in the heterogeneity of surgical indications and approaches used across the 62 participating hospitals, as well as the absence of surgeon-related variables (experience, case volume, technical preference), all of which may influence implant performance. Moreover, although all stems were tapered conical designs, implant characteristics were not uniform: differences in surface finish, metallurgy, length, and spline geometry across manufacturers may have contributed to variations in fixation and subsidence risk. Finally, the 20-year timeframe encompasses several generational changes in implant design and modular junction technology, making it likely that early-generation modular stems performed differently from modern ones. Despite these limitations, the large sample size and extended follow-up period provide robust insights into long-term survival trends and permit meaningful comparisons between monoblock and modular tapered stem designs. The core finding of our study is that monoblock conical stems used in revision THA surgery exhibit significant superior long-term survival compared with modular designs. Current literature regarding conical stems remains controversial regarding the superiority of monoblock vs. modular designs: several clinical studies compared the outcomes of these two stem designs, yet the results remain inconsistent, making it unclear which implant performs better in rTHA [12, 16, 17]. Yacovelli et al. [18], in 2021 reported the outcomes from 288 rHTA patients—225 with modular and 63 with monoblock stems— showing no significant differences in overall revision rates (5.78% for modular stems versus 9.52% for monoblocks). Similarly, Huang et al. [19], in 2017 retrospectively analyzed 160 rTHA patients managed by modular conical stems and 128 by monoblock conical stems implanted over six years; they found no differences in postoperative Harris Hip Scores, overall satisfaction, 8-year cumulative survival, or rates of infection, dislocation, and periprosthetic fracture. Feng et al. [20] in 2020 also observed comparable survival rates between modular (95.4%) and monoblock (95.5%) stems in an 8-year follow-up study involving 218 patients. Moreover, Cohn et al. [11] in 2020, in a study of 145 femoral stem revisions (67 modular vs. 78 monoblock implants) found no significant differences in reoperation rates (22.3% vs. 17.9%), intraoperative fractures (9.0% vs. 3.8%), postoperative fractures (3.0% vs. 1.3%), dislocation (11.9% vs. 5.1%), or aseptic loosening (4.5% vs. 6.4%) between the two cohorts. In contrast with these short- to medium-term studies, our long-term findings suggest that monoblock stems are associated with overall better survival. While existing literature does not provide extensive long-term retrospective analyses, our study indicates that monoblock stems in rTHA surgery have superior 20-year survival outcomes.

Despite these favourable survival rates, the tapered stem design is associated with the risk of subsidence, which can lead to implant instability, dislocation risk, and leg length discrepancy. Regarding subsidence, Yacovelli et al. [18] reported average subsidence values of 3.55 mm for modular stems and 2.44 mm for monoblock stems, with no significant differences at final follow-up; similarly, analyses with a subsidence cut-off of at least 5 mm failed to detect differences between groups. Pomeroy et al. [21] in a retrospective study of 271 revision hip arthroplasties found no significant differences in the overall subsidence (*p* = 0.191) or the incidence of subsidence >5 mm (*p* = 0.126) comparing monoblock and modular stems. Conversely, Hung et al. [19] observed that subsidence was significantly lower in the modular group (0.95 mm) compared with monoblocks (1.93 mm). Clair et al. [22] reported average subsidence values of 3.9 ± 2.6 mm for modular stems versus 2.3 ± 2.5 mm for monoblock stems, with subsidence >5 mm occurring in 29.2% of modular implants vs. 11.3% in monoblock cases. Although our registry data do not allow for a radiographic analysis of subsidence, our findings indirectly suggest a potential association between modular design and increased subsidence risk. In fact, we found that instability and dislocation were the main causes of failure for modular stems (24.7% cause of failure in this group), conditions that are strictly related with subsidence, suggesting a stronger relationship between the use of modular stems and risk of subsidence.

Over recent years, modifications in the geometry of tapered stems were developed to improve their performance, including the increase in the degree of conicity. Only small retrospective studies are available so far, and report on short term follow-up; these highlighted better outcomes for higher degree of conicity of implants. Pierson et al. [23], in a comparative study in vitro investigated how the degree of taper angle influences the initial mechanical stability of the implant, measured as implant subsidence. They found that higher degrees of taper angle were superior to lower taper angles to achieve initial mechanical stability. Albert et al. [24], investigated 23 taper stems with 3° degree of conicity, with 4 years of follow-up, used in patients with severe proximal femur bone loss (Paprosky IIIA and IIIB), reporting optimal osseointegration from distal to the proximal portion and without subsidence greater than 10 mm. However, our findings indicate that, for long-term survival, a lower conicity (2°) is preferable, because a higher conicity angle (3°) appears to be correlated with higher failure rates requiring revision surgery.

## Conclusions

In conclusion, our study demonstrates the long-term effectiveness of monoblock conical stems in THA revision, with a remarkable 20-year survival rate of 93.7%, significantly outperforming modular stems (*p* = 0.009). Notably, despite the degree of conicity was uninfluent on the outcome of revision stems in rTHAs, modular stems with 2° conicity angle achieved the best outcomes, with a 91.9% survival rate af 15 years compared with 88.0% survival rate retrieved in 3°cconicity implants (*p* < 0.001). Although both monoblock and modular stems are widely utilized in THA revisions for their mechanical advantages, clinical outcomes remain contentious. Further prospective studies may clarify the relationship between conical stem geometry, subsidence, and long-term implant performance.

## Data Availability

No datasets were generated or analysed during the current study.
